# Comparison of Fat‐to‐Muscle Ratio and Body Mass Index in Predicting Perioperative Complications and Functional Recovery After Total Knee Arthroplasty: A Prospective Cohort Study

**DOI:** 10.1111/os.70207

**Published:** 2025-11-21

**Authors:** Long Zhao, Liqun Wang, Duan Wang, Haoyang Wang, Jiali Chen, Zongke Zhou

**Affiliations:** ^1^ Department of Orthopedics and Orthopedic Research Institute West China Hospital, Sichuan University Chengdu China

**Keywords:** body mass index (BMI), fat‐to‐muscle ratio (FMR), total knee arthroplasty (TKA)

## Abstract

**Objectives:**

Obesity significantly influences the future of total knee arthroplasty (TKA). However, body mass index (BMI), the prevailing proxy for obesity, has limited predictive value for TKA outcomes, necessitating a more accurate obesity indicator. This study aimed to assess the utility of the fat‐to‐muscle ratio (FMR) in predicting postoperative outcomes related to obesity in patients undergoing TKA and compared its predictive value with that of BMI.

**Methods:**

After excluding patients with secondary osteoarthritis, severe joint deformity, or neuromotor deficits, prospective data from 146 unilateral primary TKA patients were analyzed, including demographics, BMI, and systemic and leg‐specific FMR. Primary outcomes included complications and 12‐month patient‐reported function (assessed using the University of California, Los Angeles [UCLA] activity scale and the Hospital for Special Surgery [HSS] score) and secondary outcomes including hospitalization length and surgery information were analyzed. Multivariable regression models were used to identify significant obesity‐related predictors of outcomes, with linear regression employed for continuous outcomes (UCLA activity score, HSS score) and logistic regression for binary outcomes (complications).

**Results:**

The mean BMI was 28.6 ± 4.4 kg/m^2^, systemic FMR 0.70 ± 0.23, and leg‐specific FMR 0.62 ± 0.19. Both systemic FMR (OR 1.094, *p* = 0.036) and BMI (OR 1.050, *p* = 0.015) predicted systemic complications, with FMR explaining more variance (partial *R* ^2^ = 0.134 vs. 0.088). Likewise, systemic FMR (RR, 0.950, *p* = 0.045; partial *R* ^2^ = 0.227) and leg‐specific FMR (RR, 0.922, *p* = 0.033; partial *R* ^2^ = 0.344) showed stronger associations with HSS functional score, than BMI (RR, 0.974, *p* = 0.037; partial *R* ^2^ = 0.118). Only leg‐specific FMR predicted wound complications (OR 1.063, *p* = 0.024; partial *R* ^2^ = 0.262), and HSS pain scores (RR = 0.923, *p* = 0.025; partial *R* ^2^ = 0.077). Neither FMR nor BMI was correlated with surgical duration, hospitalization, activity, or ULCA activity scores (*p* > 0.05).

**Conclusions:**

In this single‐center study, FMR demonstrated statistically stronger associations than BMI with both perioperative complications and 12‐month functional outcomes following TKA. FMR assessment may provide incremental value for preoperative risk stratification and functional outcome prediction in elective TKA.

## Graphical abstract

Our findings highlight that preoperative FMR assessment could reliably benefit surgeons in patient education and surgical timing determination, as FMR demonstrates superior predictive capacity compared to BMI for both complications and 12‐month patient‐reported outcomes following knee arthroplasty.

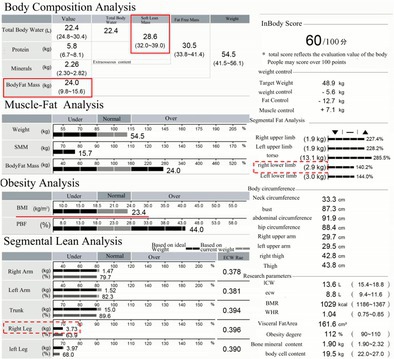

## 1 Introduction

The worldwide surge in obesity prevalence rate has reached concerning levels, with recent data revealing that nearly one‐eighth of the global population now falls into this category [1]. This epidemic carries profound implications for joint health, as the biomechanical stress and systemic effects of obesity substantially elevate the likelihood of knee osteoarthritis, thereby fueling a growing demand for total joint arthroplasty (TKA) in this demographic [2, 3, 4]. Yet, performing TKA in obese individuals remains fraught with challenges, as evidenced by prolonged surgical times, heightened infection risks, and reduced physiological resilience [5, 6]. Compounding these issues, many obese patients experience less satisfactory pain management and functional recovery postoperatively [7]. These realities highlight the important need for meticulous evaluation of obesity‐associated risks and thorough preoperative education. Conceivably, implementing a reliable, standardized tool to assess obesity‐related surgical risks could optimize patient preparation processes and improve prognosis prediction accuracy.

Body mass index (BMI) remains a prevailing proxy for obesity based on height and weight relationships. While it is highly practical for routine clinical use due to its simplicity, cost‐effectiveness, and established reference ranges, its inability to differentiate fat mass from muscle mass raises concerns regarding its sensitivity and specificity as a predictor of postoperative outcomes. Specifically, the relative imbalance between fat accumulation and muscle storage, which is undetectable by BMI (which classifies overweight as ≥ 24 kg/m^2^ and obesity as ≥ 28 kg/m^2^), was believed to independently drive metabolic dysregulation and impaired physical function [5, 8, 9]. In contrast, the fat‐to‐muscle ratio (FMR) derived from dual‐energy X‐ray absorptiometry (DEXA)—defined as the ratio of total fat mass (kg) to whole body lean mass (kg)—emerges as a physiologically grounded metric that captures the metabolic antagonism between adipose and muscle tissues, with established normal reference ranges in Chinese populations of 0.37–0.43 for men and 0.52–0.64 for women [10]. By demonstrating stronger correlations than BMI with both metabolic risk markers (including chronic inflammation and insulin resistance) and systemic functional capacity measures (including grip strength and 6‐min walking distance) [11], FMR directly addresses this critical gap. Additionally, the leg‐specific FMR variant, calculated as the ratio of leg fat mass to leg muscle mass (quadriceps + hamstrings), targets locomotor‐relevant compartments. This dual approach captures both global metabolic risk (systemic) and procedure‐specific functional implications (leg‐specific). Conceivably, this combination may provide superior predictive value for both medical metabolic complications and functional recovery following lower‐extremity procedures such as TKA.

On the one hand, studies of outcome prediction based on BMI for patients undergoing TKA often yield contradictory results in the literature [5, 8, 9]. On the other hand, research on the predictive utility of FMR for postoperative outcomes—including perioperative complications and patient‐reported functional outcomes following TKA—is lacking. Based on this gap, we designed this study to (i) analyze the relationship of both systemic and leg‐specific FMR with clinical and patient‐reported functional outcomes after TKA, and (ii) compare these with those of BMI, in a prospective cohort. Given that FMR offers a more precise and clinically relevant assessment of obesity than BMI alone, we hypothesize that these refined indicators will demonstrate superior predictive accuracy for postoperative clinical and functional outcomes following TKA.

## 2 Patients and Methods

### 2.1 Study Design

This single‐center, prospective study was conducted with approval from the Clinical Trials and Biomedical Ethics Committee of our institution (No. 2024‐92) and was registered in the Chinese Clinical Trial Registry (registration number: ChiCTR2400087646). Written informed consent was obtained from all participants prior to their inclusion in the study.

### 2.2 Patients

Patients aged 40–80 years who underwent primary unilateral TKA for Kellgren‐Lawrence grade 3–4 osteoarthritis at our institution between March and June 2024 were prospectively enrolled. Exclusion criteria included (i) rheumatoid arthritis, osteonecrosis, Charcot arthropathy, post‐traumatic or septic arthritis sequelae, (ii) knee flexion deformity of ≥ 30° or varus‐valgus deformity of ≥ 30°, (iii) cognitive impairment, neurological motor deficiencies, and (iv) unicompartmental or multiple joint arthroplasty. A priori sample size calculation was not performed; all eligible consecutive patients were enrolled.

### 2.3 Obesity Evaluation

**FIGURE 1:**
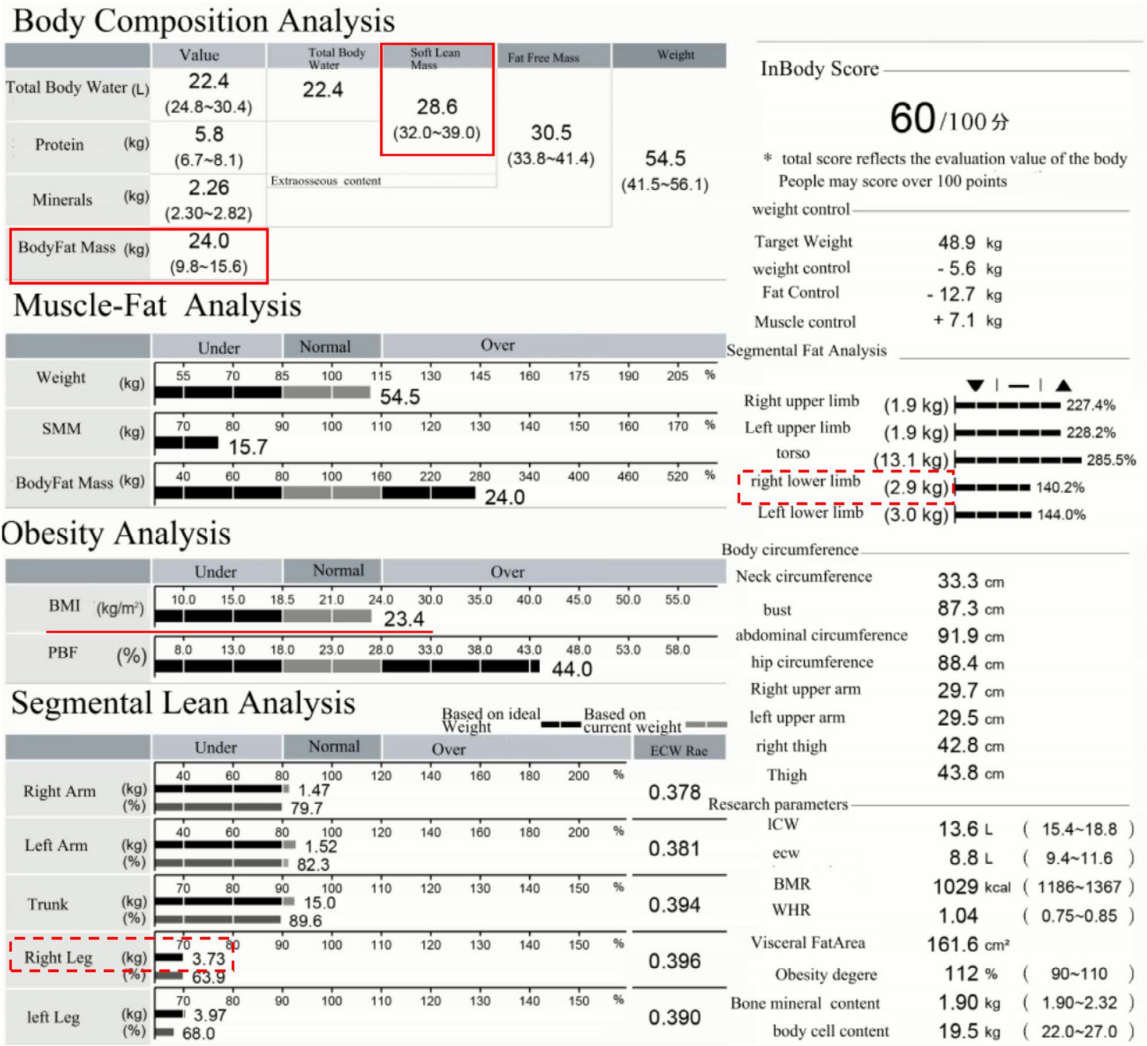
Example of a body composition report showing a significant discrepancy between body mass index (BMI) (within normal range) and soft lean mass (below normal range) with fat mass (above normal range). Solid and dashed boxes indicate systemic and leg‐specific measurements, respectively.

Preoperative height and weight measurements were obtained to calculate each patient's BMI; FMR was assessed preoperatively using the InBody770 bioelectrical impedance analyzer (BIA) (InBody Co., Seoul, Korea). All BIA measurements followed standardized protocols: (1) Preparation: 8‐h overnight fasting with bladder voiding within 30 min before testing; (2) Environmental conditions: Room temperature maintained at 22°C–24°C with 40%–60% relative humidity; (3) Timing: Conducted between 7:00 and 10:00 a.m. to minimize diurnal variation. During measurements, patients stood barefoot on the device's footplate with their feet positioned according to manufacturer guidelines, while firmly grasping the handle electrodes with 15° shoulder abduction to complete the electrical circuit. The integrated impedance analyzer measured segmental bioelectrical impedance across multiple frequencies, enabling the system's software to calculate body composition parameters through validated algorithms, including total body fat mass, lean body mass, and region‐specific skeletal muscle mass [12]. It is noteworthy that compared with traditional DEXA measurements, BIA demonstrates not only greater operational convenience and non‐invasiveness, but also strong concordance with DEXA for body composition assessment. Validation studies report correlation coefficients of 0.92 (fat mass) and 0.87 (fat‐free mass) between BIA and DEXA under standardized conditions [13]. The FMR was calculated at both systemic and leg‐specific levels: systemic FMR was determined as the ratio of total fat mass to whole‐body lean mass, while leg‐specific FMR was computed as the ratio of fat mass to skeletal muscle mass in the operative leg (Figure 1).

### 2.4 Perioperative Management

All patients underwent general anesthesia. After preoxygenation, anesthesia was induced with sufentanil (0.3 μg/kg), cisatracurium (0.2 mg/kg), propofol (2 mg/kg), and midazolam (2 mg/kg), and maintained with sufentanil (0.1 μg/kg), atracurium (0.5 mg/kg), and sevoflurane (1%–3%). The surgical procedures were performed by three fellowship‐trained arthroplasty specialists through a standardized medial parapatellar approach. The mechanical alignment technique incorporated measured resection for femoral component positioning with a gap‐balancing technique to optimize soft‐tissue tension. Femoral preparation utilized an intramedullary guide, while tibial resection employed an extramedullary alignment system. All patients received posterior‐stabilized cemented prostheses, with patellar resurfacing not performed. A pneumatic tourniquet, at 240 mmHg of pressure, was applied starting from the initial skin incision and continuing until wound closure. Oral imrecoxib (100 mg twice daily) and flurbiprofen transdermal patches (40 mg twice daily) were prescribed for postoperative analgesia. All patients were managed under enhanced recovery after surgery (ERAS) guidelines, including once intravenous cefazolin (1 g) administered 30 min pre‐anesthesia for infection prophylaxis; thromboembolism prevention with subcutaneous enoxaparin sodium (0.4 mL, 4000 IU) every 24 h starting 8 h postoperatively for 48 h, followed by oral rivaroxaban (10 mg) daily from postoperative days 3 to 14. A standardized rehabilitation protocol was initiated on postoperative day 1, comprising supine straight leg raises, walker‐assisted ambulation training, and active knee flexion exercises targeting ≥ 90° range. Discharge criteria required VAS pain ≤ 3, independent ambulation ≥ 20 m, knee flexion ≥ 90° with extension lag ≤ 10°, and absence of infection or bleeding.

### 2.5 Data Collection

Reviewed data included baseline clinicodemographic characteristics (including age, sex, American Society of Anesthesiologists [ASA] score, BMI, and FMR). Assessments were performed at hospitalization, 1, 3, 6, and 12 months postoperatively, with evaluators blinded to participants' FMR and BMI data. The primary outcome was the HSS knee score at 12 months postoperatively, which assesses pain, range of motion, and function, with excellent internal consistency (Cronbach's *α* = 0.89) and test–retest reliability (ICC = 0.91) [14]. Secondary outcomes included University of California Los Angeles (UCLA) activity score at 12‐month postoperative follow‐up (assessing patient‐reported function across 10 levels, from completely inactive [level 1] to regular sports participation [level 10]) [15], surgery duration (skin incision to closure), bleeding [(preoperative—postoperative hematocrit) × weight (kg) × 0.08], hospitalization days (admission to discharge), wound complications (superficial/deep surgical site infection, and impaired healing), and systemic complications. The latter were defined as any of the following occurring within 30 postoperative days: liver dysfunction (ALT > 3 times the upper limit of normal); cardiac dysfunction (NT‐proBNP > 300 pg/mL or arrhythmia); respiratory infection (radiographic pneumonia with fever > 38°C or leukocytosis > 12,000/μL); urinary tract infections (UTI) (dysuria or urgency with positive urine culture); or noninfectious fever (temperature ≥ 38.3°C twice ≥ 4 h apart without infection evidence).

### 2.6 Statistical Analysis

Descriptive statistics were computed for all variables, with continuous variables expressed as mean ± standard deviation and categorical variables as frequencies (percentages). Multivariable generalized linear models (linear regression for continuous outcomes; logistic regression for dichotomous outcomes) were used to examine associations between obesity indices (BMI and FMR) and outcomes, adjusted for prespecified covariates (sex, age, ASA scores). For dichotomous outcomes (e.g., complications), logistic regression generated odds ratios (ORs) with 95% confidence intervals (CIs). Continuous outcomes (HSS scores, UCLA activity scores, hospitalization duration) were analyzed using linear regression, reporting risk ratios (RRs) with 95% CIs. The normality of continuous variables was confirmed by Shapiro–Wilk tests (all *p* > 0.10). Decile‐based risk stratification identified inflection points, subsequently validated by ROC analysis with optimal thresholds determined by maximizing the Youden index. Model fit was assessed using Partial *R* ^2^ (*R* ^2^), and linearity assumptions were verified via Harrell's restricted cubic spline test (all *p* > 0.05). Type I error was controlled by Bonferroni correction. All analyses were conducted using SPSS (Version 29.0.1, IBM Corp, Armonk, NY), with two‐tailed *p* < 0.05 considered statistically significant.

## 3 Result

### 3.1 General Clinical‐Demographic Results

**FIGURE 2:**
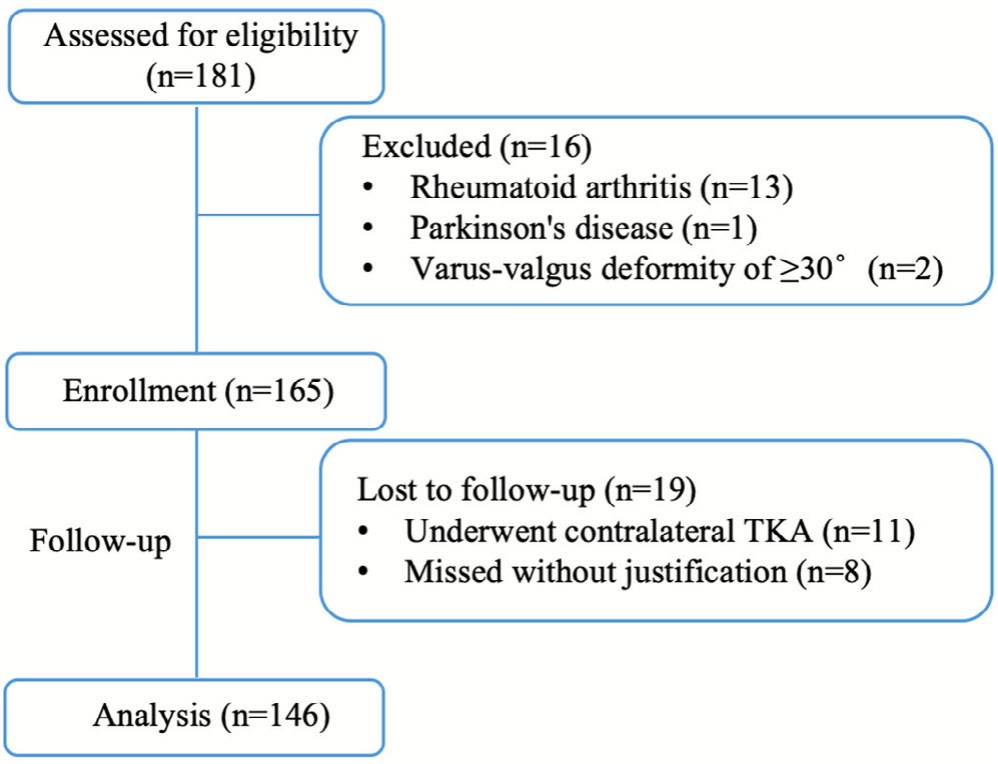
Flowchart of patient screening and follow‐up.

A total of 165 consecutive patients were initially enrolled. Of these, 19 patients were lost to follow‐up and excluded from analysis because their baseline characteristics were comparable to the retained cohort (indicating random missingness), resulting in a final analytical cohort of 146 patients (Figure 2; Table 1).

**TABLE 1:** Patient baseline clinicodemographic characteristics.

Characteristics	Outcomes
Age (years)^a^	67.1 (7.4)
Sex (male: female)^b^	35:111
Height (cm)^a^	157.0 (9.1)
Weight (kg)^a^	74.4 (13.6)
BMI (Kg/m^2^)^a^	28.6 (4.4)
Systemic FMR^a^	0.70 (0.23)
Leg‐specific FMR^a^	0.62 (0.19)
ASA score^b^	
I	10
II	103
III	33

^a^ The values are presented as mean (standard deviation).

^b^ The values are presented as the number (percentage) of patients.

### 3.2 Prediction in Post‐TKA Medical Complications

In multivariable analyses, both systemic FMR (OR, 1.094; 95% CI, 1.002 to 1.891; *p* = 0.036) and BMI (OR, 1.050; 95% CI, 1.020 to 1.302; *p* = 0.015) significantly predicted systemic complications, including liver dysfunction (*n* = 8), cardiac dysfunction (*n* = 5), respiratory infections (*n* = 2), urinary tract infections (*n* = 2), and postoperative non‐infective fever (*n* = 7). Systemic FMR explained more variance (partial *R* ^2^ = 0.134) than BMI (partial *R* ^2^ = 0.088). Each one‐standard deviation increase in systemic FMR was associated with a 9.4% higher risk of complications, which was approximately twice that of BMI (5.0% risk increase per one‐standard deviation higher BMI). Only leg‐specific FMR predicted wound issues (impaired healing [*n* = 5], superficial surgical site infection [*n* = 3]) (OR, 1.063; 95% CI, 1.018 to 1.277; *p* = 0.024; partial *R* ^2^ = 0.262), with each one‐standard deviation increase in regional FMR associated with a 6.3% higher complication risk (Table 2). Neither systemic nor leg‐specific FMR, nor BMI predicted surgical duration, blood loss, or hospital stay (Table 3).

**TABLE 2:** Prediction of dichotomous clinical outcomes after adjustment for significant baseline demographic covariates.

Outcome and predictor	Outcomes^a^	Odds ratio^b^	*R* ^2^	*p*
Systemic complications				
BMI	24:122	1.050 (1.020–1.302)	0.088	0.015^c^
Systemic FMR		1.094 (1.002–1.891)	0.134	0.036^c^
Leg‐specific FMR		1.123 (0.684–2.984)	—	0.089
Wound complications				
BMI	8:138	1.240 (0.555–3.846)	—	0.258
Systemic FMR		0.970 (0.840–1.952)	—	0.107
Leg‐specific FMR		1.063 (1.018–1.277)	0.262	0.024^c^

^a^ Data are presented as the ratio of “yes” to “no” outcomes.

^b^ Values represent odds ratios per one standard deviation increase in the covariate, accompanied by 95% confidence intervals in parentheses.

^c^ Indicates statistical significance at *p* < 0.05 level.

**TABLE 3:** Prediction of count data for clinical and patient‐reported functional outcomes after adjustment for significant baseline demographic covariates.

Outcome and predictor	Outcomes^a^	Risk ratio^b^	*R* ^2^	*p*
Surgical time (minutes)				
BMI	84.00 (26.40)	1.453 (1.194–3.100)	—	0.348
Systemic FMR		0.844 (0.457–7.884)	—	0.487
Leg‐specific FMR		1.881 (1.057–2.653)	—	0.741
Surgical bleeding (mL)				
BMI	75.88 (49.41)	0.867 (0.452–9.388)	—	0.497
Systemic FMR		1.171 (1.091–1.478)	—	0.210
Leg‐specific FMR		0.879 (0.541–10.325)	—	0.117
Hospitalization (days)				
BMI	5.77 (1.40)	1.440 (0.900–2.787)	—	0.240
Systemic FMR		1.521 (0.741–4.213)	—	0.345
Leg‐specific FMR		1.674 (1.211–6.387)	—	0.121
UCLA activity score				
BMI	4.21 (1.34)	0.971 (0.643–1.813)	—	0.348
Systemic FMR		0.881 (0.705–1.620)	—	0.172
Leg‐specific FMR		0.864 (0.674–1.312)	—	0.094
HSS function score				
BMI	17.62 (3.20)	0.974 (0.954–0997)	0.118	0.037^c^
Systemic FMR		0.950 (0.892–0.984)	0.227	0.045^c^
Leg‐specific FMR		0.922 (0.845–0.964)	0.344	0.033^c^
HSS pain score				
BMI	25.47 (3.47)	0.681 (0.442–0.874)	—	0.281
Systemic FMR		0.871 (0.744–1.201)	—	0.088
Leg‐specific FMR		0.923 (0.872–0.970)	0.077	0.025^c^

^a^ The values are given as mean (standard deviation).

^b^ The values are given as the risk ratio per one standard deviation increase in the covariate, with the 95% confidence interval in parentheses.

^c^ Predictor was significant at *p* < 0.05.

### 3.3 Prediction in PostTKA Functional Outcomes

In the analysis of postoperative HSS function scores, both leg‐specific and systemic FMR, as well as BMI, emerged as significant predictors. Systemic FMR (RR, 0.950; 95% CI, 0.892–0.984; *p* = 0.045; partial *R* ^2^ = 0.227) and leg‐specific FMR (RR, 0.922; 95% CI, 0.845–0.964; *p* = 0.033; partial *R* ^2^ = 0.344) had greater explanatory power than BMI (RR, 0.974; 95% CI, 0.954–0.997; *p* = 0.037; partial *R* ^2^ = 0.118). Each one‐standard deviation increase in leg‐specific and systemic FMR was associated with reductions of 5.0% and 7.8%, respectively, in HSS function scores—approximately 2–3 times greater than the reduction associated with BMI (2.6% per one‐standard deviation increase). Notably, only leg‐specific FMR significantly predicted HSS pain score (RR = 0.923; 95% CI, 0.872 to 0.970; *p* = 0.025; partial *R* ^2^ = 0.077), indicating a 7.7% reduction in HSS pain scores per one‐standard deviation increase in leg‐specific FMR. Additionally, a trend toward significance was observed for leg‐specific FMR in predicting UCLA activity scores (RR = 0.864; 95% CI, 0.674 to 1.312; *p* = 0.094), whereas neither systemic FMR nor BMI showed an association (Table 3).

### 3.4 Potential Cutoff Values for Obesity Indicators

**FIGURE 3:**
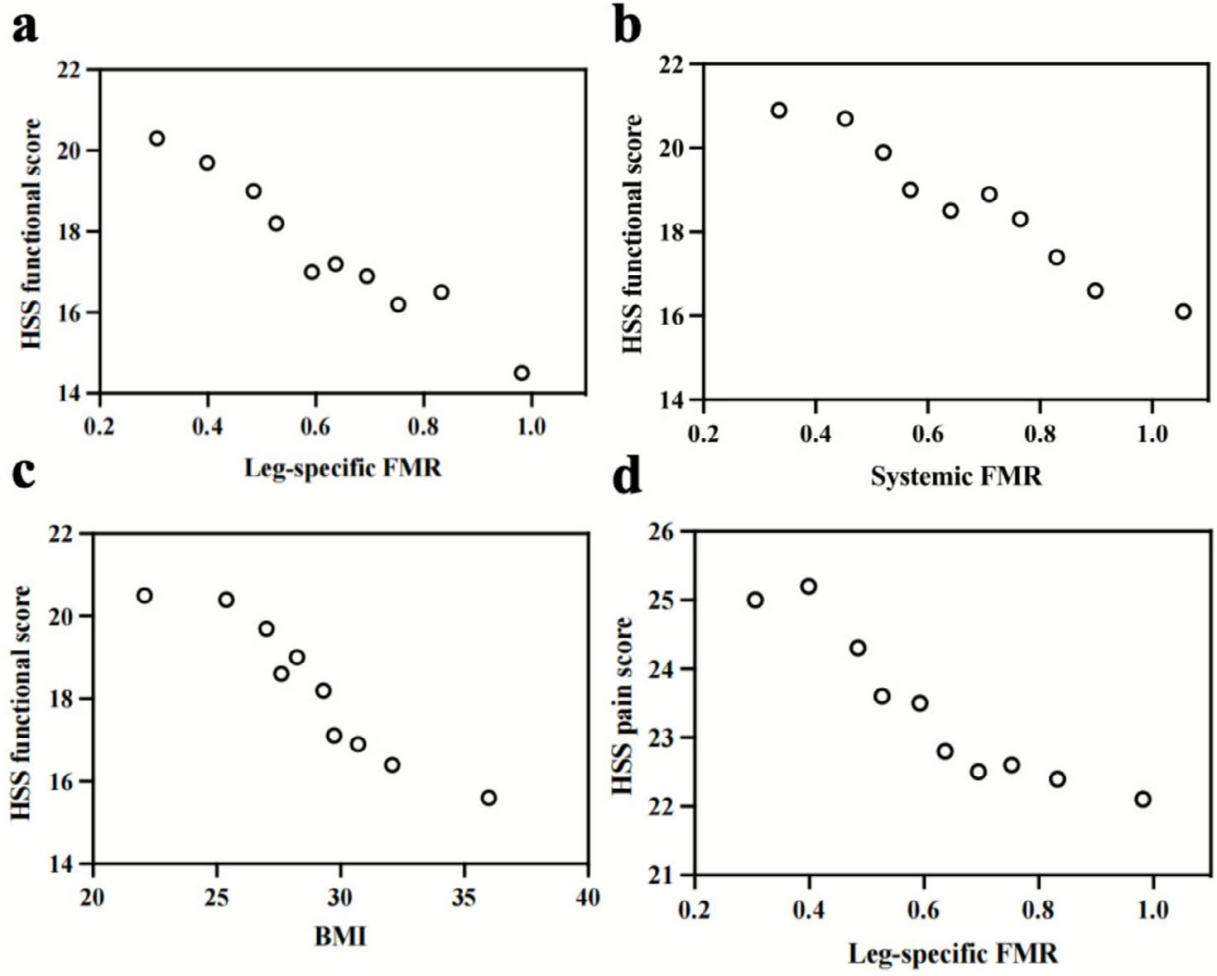
The Hospital for Special Surgery (HSS) function score showed significant negative correlations with leg‐specific fat‐to‐muscle ratio (FMR) (*r* = −0.208) (a), systemic FMR (*r* = −0.203) (b), and BMI (*r* = −0.137) (all *p* < 0.001) (c). HSS pain scores also correlated negatively with leg‐specific FMR (*r* = −0.186, *p* < 0.001) (d). Scatter plot stratified by deciles: Data points are grouped according to FMR or BMI deciles (*N* = 146).

Additional analyses were conducted to identify significant predictors of specific clinical outcomes and potential cutoff values for obesity indicators. Patients with systemic FMR > 0.94 (OR = 3.110; 95% CI, 1.737 to 18.864; *p* = 0.024) or BMI > 35.5 (OR = 2.354; 95% CI, 1.143 to 9.734; *p* = 0.033) had significantly higher odds of systemic complications. Similarly, the risk of wound complications increased significantly when leg‐specific FMR exceeded 1.00 (OR = 4.563; 95% CI, 1.274 to 24.653; *p* = 0.040). The HSS functional score showed a negative correlation with leg‐specific FMR (*r* = −0.208, *p* < 0.001), and a leg‐specific FMR > 0.92 predicted a significantly reduced risk ratio (RR = 0.573, 95% CI, 0.154 to 0.912; *p* < 0.001) (Figure 3a). Although definitive predictive thresholds were not established, significant inverse correlations were observed between systemic FMR (*r* = −0.203, *p* < 0.001) (Figure 3b) and BMI (*r* = −0.137, *p* < 0.001) (Figure 3c) with HSS function scores, and between leg‐specific FMR and HSS pain scores (*r* = −0.186, *p* < 0.001) (Figure 3d).

## 4 Discussion

To our knowledge, this study is the first to introduce systemic and leg‐specific FMR as alternative obesity indicators and systematically compare their predictive performance against the traditional BMI in a prospective cohort design. Our findings highlight that FMR exhibited statistically stronger associations compared to BMI, especially in forecasting both clinical outcomes, including systemic complications and wound issues, as well as 12‐month patient‐reported functional outcomes following TKA.

### 4.1 The Better Predictiveness of FMR for Medical Complications After TKA

Clinically, obesity exhibits substantial heterogeneity that frequently challenges conventional BMI‐based classifications. Mounting evidence reveals striking variations in body composition among individuals with identical BMIs, including up to twofold differences in adiposity and 30 kg disparities in lean mass [16]. These limitations stem from BMI's fundamental inability to distinguish between two physiologically distinct compartments—fat mass and lean body mass [17]. In contrast, FMR quantifies these components separately, providing superior clinical discrimination. This advantage endows FMR with dual capacity: elevated FMR captures adipose‐driven metabolic dysfunction (where visceral fat deposition, releases free fatty acids that induce hepatic insulin resistance, increasing risks of hyperglycemia‐related infections and renal injury) and muscle‐mediated reserve depletion (where low muscle mass, limits amino acid availability for acute‐phase protein synthesis, impairing stress response), thus more accurately predicting postoperative systemic complications following abdominal surgery [18, 19]. Our findings corroborate this pattern in TKA patients: each 1‐SD increase in systemic FMR was associated with a 9.4% greater risk of medical complications—nearly double the risk linked to an equivalent BMI increase (5.0%), though caution is needed when interpreting these coefficients, given the divergent clinical implications of a 1‐SD change in BMI versus FMR. Contemporary evidence highlights that increased local subcutaneous adipose thickness is a well‐validated predictor of elevated wound complication risk [20, 21]. Our results substantiate these observations, demonstrating that elevated leg‐specific FMR independently predicted wound issues, with each standard deviation increase conferring a 6.3% higher risk after BMI adjustment. This association likely arises from: (1) insufficient nutrient delivery and impaired immune cell trafficking (due to reduced capillary density from intramuscular fat infiltration); (2) increased myosteatosis‐mediated inflammatory factors; and (3) weakened mechanical support for tissue healing (due to muscle loss) [22, 23]—both hallmark features of elevated leg‐specific FMR. These findings collectively advocate for incorporating FMR, rather than relying solely on BMI, into preoperative risk stratification for total knee arthroplasty patients.

### 4.2 The Superior Predictiveness of FMR for Functionality After TKA

Beyond metabolic risk, FMR shows particular value in predicting physical function status in previous studies. Growing evidence establishes FMR as a more sensitive metric than traditional obesity measures (BMI, waist circumference) for assessing muscle strength decline and physical performance deficits, including grip strength, gait parameters, and walking endurance [24, 25]. Our cohort analysis demonstrates this relationship persists in patients following TKA, with leg‐specific FMR showing superior predictive value for functional recovery outcomes. Quantitatively, each standard deviation increase in leg‐specific FMR corresponded to a 7.8% reduction in HSS function scores—a threefold greater effect than the 2.6% decrease associated with BMI increases. This predictive superiority stems from FMR's direct quantification of the critical muscle‐fat balance. Elevated leg‐specific FMR typically indicates myosteatosis or pathological intramuscular fat infiltration, which compromises quadriceps function through multiple mechanisms: structural impairment of contractile architecture and mitochondrial dysfunction [26, 27]; metabolic disturbance from adipose‐derived pro‐inflammatory cytokines (TNF‐α, IL‐6) [28, 29]; and reduced joint stabilization capacity. These interconnected pathways explain why leg‐specific FMR outperforms BMI in forecasting postoperative functional recovery, providing clinicians with a more nuanced tool for preoperative assessment and personalized rehabilitation planning in TKA patients. Although sarcopenia and body fat percentage (BFP) have established prognostic value in arthroplasty populations [30, 31], future studies should conduct systematic head‐to‐head comparisons of body composition metrics (specifically FMR vs. sarcopenia indices vs. BFP) to determine their relative predictive performance.

### 4.3 Limitations and Strengths

Several limitations must be acknowledged when interpreting these findings. First, this single‐center study focused on osteoarthritis patients undergoing TKA with a short‐term follow‐up period. No formal a priori sample size calculation was performed due to the exploratory nature of this clinical cohort analysis. These methodological considerations may limit the generalizability of our findings to broader TKA populations and reduce statistical power to detect smaller effects. Future studies should validate these results in more diverse clinical populations with longer follow‐up periods, incorporating formal sample size calculations based on primary outcomes. Second, while focusing on mild‐to‐moderate obesity (BMI 30–39.9 kg/m^2^), future studies should include the complete obesity severity spectrum (BMI: 25–29.9 kg/m^2^ and ≥ 40 kg/m^2^) to validate these findings across all patient groups. Third, although we controlled for key demographic and clinical confounders including sex, age, and ASA scores, potential residual confounding from unmeasured variables such as socioeconomic status and activity levels remains possible. Finally, our reliance on single baseline FMR measurements prevents evaluation of longitudinal body composition changes—an important consideration for future research examining temporal variations in body composition relative to TKA outcomes. Finally, potential barriers to implementation of BIA—including the devices’ cost, the need for regular calibration and maintenance, and staff training—may limit adoption in grassroots institutions.

Nevertheless, our study also has notable strengths. To our knowledge, this is the first investigation to comprehensively compare the predictive utility of FMR and BMI specifically within a TKA population, providing novel insights into optimal obesity assessment in this surgical context.

## 5 Conclusions

In this single‐center study, FMR demonstrated statistically stronger associations than BMI with both perioperative complications and 12‐month functional outcomes following TKA, suggesting that FMR assessment provides incremental value for preoperative risk stratification. Future research should prioritize multicenter validation studies across diverse healthcare settings and populations with varying comorbidities (e.g., diabetes, osteoporosis), along with longitudinal evaluation of FMR changes in relation to long‐term implant survivorship. For clinical implementation, we propose integrating FMR assessment into the standard preoperative workflow concurrently with anthropometric measurements, enabling targeted prehabilitation for high‐risk patients defined by FMR thresholds.

## Author Contributions

**Long Zhao:** conceptualization, writing – original draft. **Liqun Wang** and **Haoyang Wang:** methodology, data curation. **Duan Wang:** validation, supervision. **Jiali Chen** and **Zongke Zhou:** project administration, supervision.

## Disclosure

All authors listed meet the authorship criteria according to the latest guidelines of the International Committee of Medical Journal Editors, and all authors are in agreement with the manuscript.

## Ethics Statement

This study was performed in line with the principles of the Declaration of Helsinki. Approval was granted by the Ethics Committee of West China Hospital, Sichuan University (Approval No. 2024–92).

## Consent

Informed consent was obtained from all individual participants included in the study. The study was registered in the Chinese Clinical Trial Registry (registration number: ChiCTR2400087646).

## Conflicts of Interest

The authors declare no conflicts of interest.

## Data Availability

The data that support the findings of this study are available on request from the corresponding author. The data are not publicly available due to privacy or ethical restrictions.
